# Study on the predictive value of femoral head HU values in hip fractures

**DOI:** 10.3389/fsurg.2026.1862768

**Published:** 2026-07-02

**Authors:** Wenjie Qin, Jiangyun Liao, Jianwen Cheng

**Affiliations:** 1 Department of Orthopaedics Trauma and Hand Surgery, The First Affiliated Hospital of Guangxi Medical University, Nanning, Guangxi, China; 2 Department of Operating Room of Anesthesiology, Fourth Affiliated Hospital of Guangxi Medical University/Liuzhou City Worker Hospital, Liuzhou, Guangxi, China

**Keywords:** bone density, fracture fixation, hip fractures, Hounsfield units, osteoporosis

## Abstract

**Objective:**

This study investigates whether the CT Hounsfield Unit (HU) value of the femoral head can differentiate femoral neck fractures (FNF) from intertrochanteric femoral fractures (IFF), and whether it can serve as an indicator for predicting the complexity of intertrochanteric fractures.

**Methods:**

A retrospective analysis was conducted on 234 patients (117 with FNF and 117 with IFF), measuring the average HU value of the femoral head on preoperative CT. According to the Orthopaedic Trauma Association/AO Foundation (AO/OTA) classification, IFFs were categorized into stable (A1.1–A2.1) and unstable (A2.2–A3) types. Statistical comparisons were performed using independent sample *t*-tests, chi-square tests, and analysis of variance.

**Results:**

HU values could not distinguish FNF from IFF, but within IFFs, unstable fractures had significantly lower HU values than stable ones. Male patients had significantly higher HU values than females. ROC analysis suggested that HU values showed high specificity (91.89%) but low sensitivity (17.5%) for predicting intertrochanteric fracture stability, with moderate overall diagnostic performance (AUC = 0.6764). Gender-specific thresholds were identified but also showed moderate performance. These values support a rule-in role rather than general screening.

**Conclusion:**

Femoral head HU values are a reliable imaging marker for predicting intertrochanteric fracture stability and identifying high-risk patients, particularly women with unstable fractures, with consideration for sex differences in application.

## 1 Introduction

With the intensification of societal ageing, hip fractures have become a common injury posing a serious threat to elderly health. Their high mortality and disability rates impose a heavy burden on patients' families and society (1). Research indicates that mortality within one year of hip fracture can reach 20%–30% (2), with its occurrence closely linked to osteoporosis (3). Bone mineral density (BMD) serves as a core indicator for assessing fracture risk, with dual-energy x-ray absorptiometry (DEXA) widely employed as the gold standard for its measurement (4, 5). However, DEXA is primarily applied to axial bones, presenting limitations when evaluating peripheral bones such as the hip (6). Computed tomography (CT) provides high-resolution skeletal imaging and enables bone density measurement, offering a novel technical approach for hip assessment. FNF and IFF represent the most prevalent types of hip fractures. Postoperative functional recovery is often suboptimal, particularly in IFF patients who frequently experience residual functional impairment (7), a phenomenon associated with fracture type and healing outcomes (8). Consequently, the capacity to predict both the type and severity of fractures through early bone density assessment carries profound clinical significance for both prevention strategies and management approaches. The quality of bone in the femoral head may exhibit significant correlations with the occurrence and progression of hip fractures. This study seeks to explore the potential utility of HU values as a predictive marker for different types of hip fractures. Specifically, we aim to compare HU values within the femoral head between patients diagnosed with IFF and those with FNF, as observed on CT scans. Furthermore, it seeks to evaluate the predictive capability of HU values for IFF severity, thereby providing novel reference criteria for clinical assessment.

## 2 Materials and methods

### 2.1 Study cohort

Review of medical records from January 2022 to December 2024 was conducted for patients aged ≥65 years with either femoral neck or intertrochanteric fractures. As this is a retrospective study, informed consent was waived according to the approval of our hospital's Ethics Review Committee. Inclusion criteria required patients to have undergone three-dimensional dual-hip CT scanning within our healthcare institution. Exclusion criteria were: (1) high-energy trauma (defined as motor vehicle accidents or falls from ≥2 metres height) (9); (2) CT scans acquired from other institutions; (3) previous surgical intervention or focal pathologies—such as avascular necrosis of the femoral head, arthritis, or bone cysts—that would render measurements unreliable. Ultimately, 234 patients met inclusion criteria (see Table 1). In this study, fractures classified as AO/OTA 31-A1.1–31-A2.1 were defined as stable intertrochanteric fractures, characterized by intact posteromedial cortical bone, mild comminution and good mechanical stability. Fractures categorized as AO/OTA 31-A2.2–31-A3 were defined as unstable intertrochanteric fractures, which were accompanied by obvious posteromedial cortical bone disruption, large bone defect, severe comminution and poor inherent mechanical stability, easily leading to postoperative displacement and internal fixation failure. This study aims to provide novel reference data. At our institution, CT examination is routinely performed for all elderly patients with suspected hip fracture as part of the standard preoperative evaluation. Therefore, all consecutive patients meeting the age and fracture criteria underwent CT scanning during admission. This study included all eligible patients with complete CT data, minimizing selection bias related to selective CT use.

**Table 1:** Patient demographics and HU values.

Variable	IFF	FNF	Value
	117	117	
Age (yrs)	79.56 ± 8.40	77.82 ± 8.31	0.113
BMI (kg/m^2^)	26.5 ± 5.4	24.7 ± 4.5	2.77
Gender			
Male	54	52	0.793
Female	63	65	
Diabetes mellitus	32 (27.4%)	35 (29.9%)	0.664
Tobacco use	21 (17.9%)	25 (21.4%)	0.510
Use of anti-osteoporosis drugs	22 (18.8%)	18 (15.4%)	0.487
HU value (mean ± SD)	182.91 ± 38.97	180.82 ± 28.69	0.467

### 2.2 Imaging data acquisition and measurement

All patients underwent imaging using a 64-slice spiral CT scanner (SIE-MENS, Germany). The scan range extended from the superior iliac crest to 5 cm below the lesser trochanter. Scan parameters: 120 kV, 350 mA, slice thickness 1.0 mm, slice spacing 0.6 mm. Patients were positioned supine; no intravenous contrast agent was administered. HU measurements were calculated on the GE Healthcare Picture Archiving and Communication System (PACS). To avoid interference from fracture lines, all HU measurements were performed on the unaffected contralateral femoral head, based on the assumption of symmetric bone density between sides.

**Figure 1:**
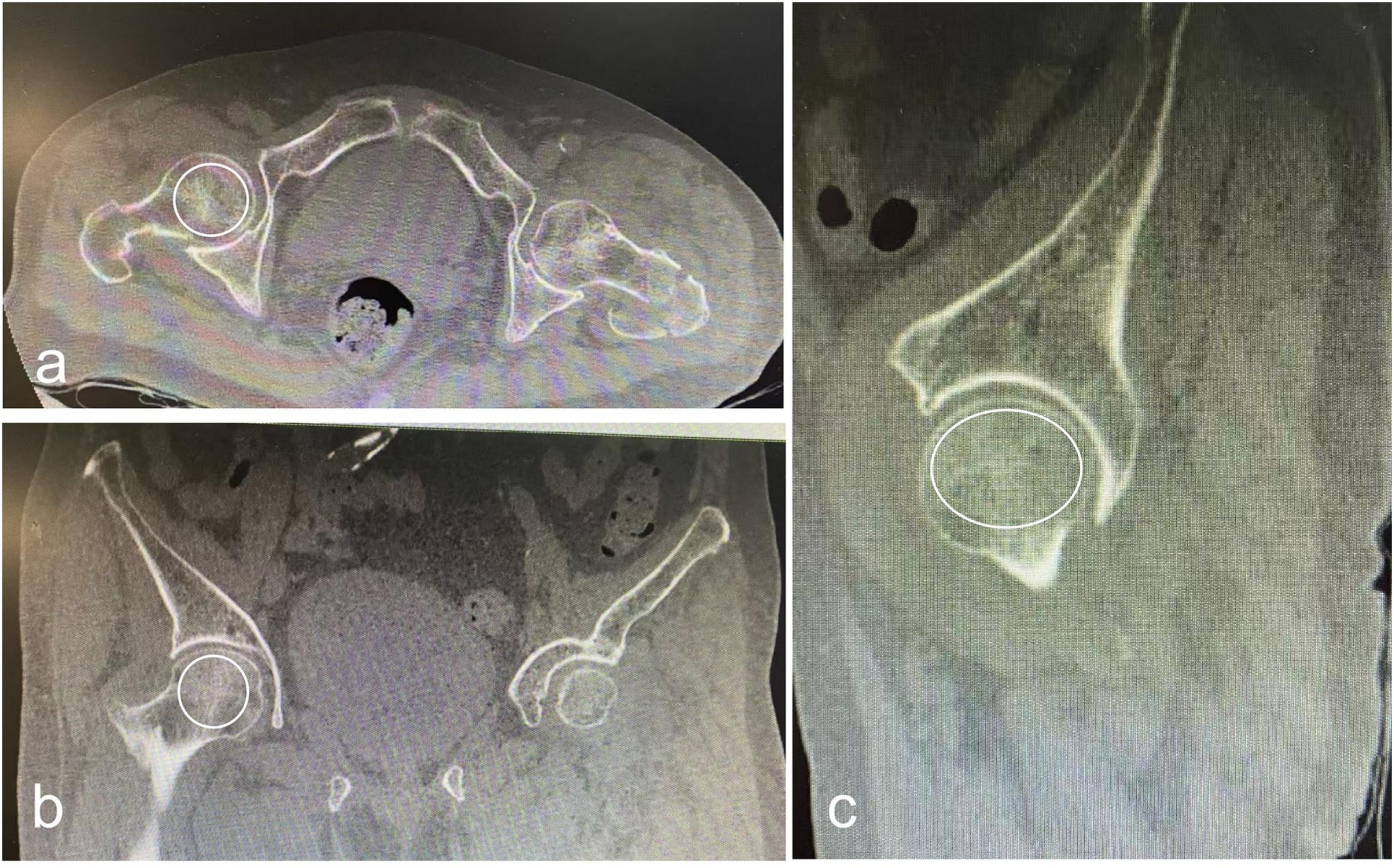
Illustrates the process of calculating the mean Hounsfield unit (HU) value of the contralateral femoral head. Examples of regions of interest (ROIs) for Hounsfield units are shown on three-phase computed tomography (CT) images of the femoral head. Axial position: **(a)** 148.29 HU, coronal position: **(b)** 199.90 HU, sagittal position: **(c)** 193.58 HU.

Parameter measurement and reliability testing: Measurements were performed jointly by an orthopaedic surgeon and a senior radiologist. Manually placed circular regions of interest (ROIs) of maximum possible size were positioned at the centre of the femoral head's largest cross-sectional area in the transverse, sagittal, and coronal planes. This can be confirmed through the dynamic navigation tool of the PACS software. ROIs placement adhered to the following principles: a) complete confinement within cancellous bone; b) uniform distance from cortical margins to minimise partial volume effects; c) Avoid any macroscopically visible trabecular gaps, vascular grooves, or areas of cystic degeneration. The mean HU value of the three ROIs was recorded, and their arithmetic mean was taken as the final HU value for that femoral head (Figure 1), consistent with previous studies (10–12).

### 2.3 Inter-rater and intra-rater reliability assessment

To assess the reliability of the Henich unit measurement, 30 CT images (approximately 13%) were randomly selected for reliability analysis. The same images were remeasured by Examiner A at least two weeks apart (intra-examiner reliability), and independently measured by Examiner B (inter-examiner reliability). The intra-class correlation coefficient (ICC) and its 95% confidence interval were calculated using a two-way mixed-effects model, absolute agreement, single measurement ICC (ICC 3,1) to evaluate measurement reliability, with ICC > 0.75 indicating good consistency and ICC > 0.90 indicating excellent consistency. Intra-rater reliability was excellent with an ICC of 0.97 (95% CI: 0.94–0.99). Inter-rater reliability was also excellent with an ICC of 0.95 (95% CI: 0.91–0.98).

### 2.4 Statistical analysis

Statistical analysis was performed using SPSS 22.0. Continuous variables were presented as mean ± standard deviation. The Shapiro–Wilk test was firstly used to verify the normal distribution of data. Independent-samples *t*-test was adopted for comparisons between two groups with normal distribution. Categorical variables were presented as frequency (percentage) and analysed using chi-square tests. Pearson correlation analysis assessed the relationship between age and HU values, while receiver operating characteristic curves evaluated the ability of HU values to distinguish fracture status. Intra-rater reliability was assessed using the ICC (3,1) model, while inter-rater reliability employed the ICC (2,1) model. Statistical significance was set at *p* < 0.05. The optimal cutoff value for the ROC analysis was determined using the Youden index (sensitivity + specificity−1) to maximize the overall diagnostic accuracy. The minimum distance to the top-left corner was also evaluated for confirmation.

## 3 Results

This study included 234 patients, comprising 117 cases of IFF and 117 cases of FNF. There were no statistically significant differences between the two groups in baseline characteristics including age, body mass index, gender, diabetes, smoking status, and use of anti-osteoporosis medication (*P* > 0.05), as shown in Table 1.

Measurement reliability analysis demonstrated excellent intra and inter-rater consistency (ICC values of 0.97 and 0.95 respectively) (13). No significant difference was observed between groups in femoral head HU values (*P* > 0.05), as shown in Table 2.

**Table 2:** Intra- and inter-observer reliability of femoral head HU measurements.

Measurement reliability	ICC (3,1)	95% CI	*P*-value
Intra-rater reliability	0.97	0.94–0.99	<0.001
Inter-rater reliability	0.95	0.91–0.98	<0.001

In the IFF subgroup analysis, the HU values for unstable fracture patients (*n* = 80) (174.52 ± 34.54 HU) were significantly lower than those for stable fracture patients (*n* = 37) (201.04 ± 41.75 HU) (*p* < 0.05), as shown in Table 3. The overall HU value for male patients (194.18 ± 37.10 HU) was significantly higher than that for female patients (173.25 ± 37.93 HU) (*p* = 0.003), with a mean difference of 20.93 HU (95% CI: 7.53–34.33; t = 3.062, *p* = 0.003). The effect size for this difference was moderate (Cohen's d = 0.56). This difference remained significant in the unstable fracture subgroup (males: 186.93 ± 32.66 HU; females: 165.35 ± 32.98 HU; *p* < 0.01), whereas no statistically significant difference was observed in the stable fracture subgroup, as shown in Table 4.

**Table 3:** Classification comparison of intertrochanteric fractures of the femur.

Variable	Fracture type	
	Stable fracture (A1.1–A2.1)	Unstable fracture (A2.2–A3)
Cases	37	80
HU value(mean ± SD)	201.04 ± 41.75	174.52 ± 34.54
t-value	3.610	
*P*-Value	<0.01	

**Table 4:** HU value by gender and fracture stability in intertrochanteric fractures.

Variable	Cases	Fracture type (mean ± SD)		Total (mean ± SD)
		Stable fracture	Unstable fracture	
Male	54 (21/33)	213.20 ± 38.90	186.93 ± 32.66	194.18 ± 37.10
Female	63 (17/46)	194.62 ± 41.97	165.35 ± 32.98	173.25 ± 37.93
t-value	-	1.413	2.880	3.062
*P*-value	-	>0.05	<0.01	<0.01

### 3.1 Diagnostic efficacy of femoral HU values for stability of intertrochanteric fractures

**Figure 2:**
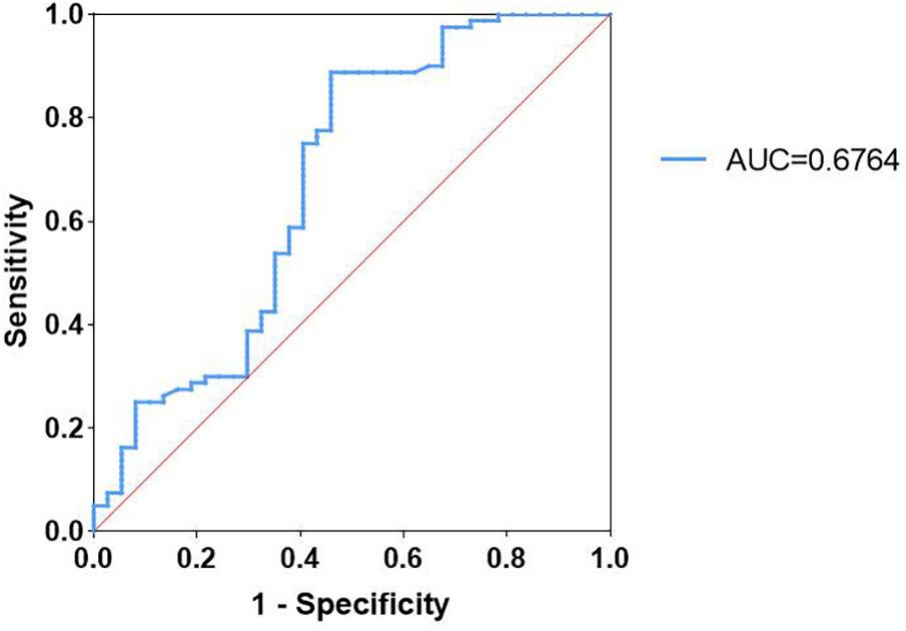
ROC curve to identify stable or unstable fractures based upon HU value (area under the curve = 0.6764). The optimal cut-off value (circle in figure) for femoral head HU value was 143.5 HU (sensitivity 0.175, specificity 0.9189).

The discriminatory capacity of femoral head Hounsfield unit values for intertrochanteric fracture stability was determined via ROC curve analysis. The optimal cutoff value was defined using the Youden index, yielding an overall cutoff of 143.5 HU. At this threshold, specificity was 91.89% and sensitivity was 17.50%, with an area under the curve (AUC) of 0.6764 (95% CI: 0.5609–0.7918). This high specificity was accompanied by low sensitivity, indicating that approximately 82% of unstable fractures would be missed at this threshold, reflecting an inherent sensitivity–specificity trade-off. See Figure 2.

**Figure 3:**
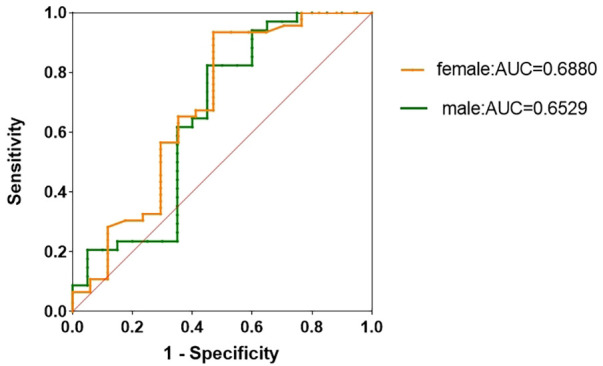
The ROC curve to distinguish between stable or unstable fractures in different genders. The AUC value was 0.65 in male patients (*n* = 54) and 0.69 in female patients (*n* = 63). Corresponding optimal cutoff values and diagnostic parameters were marked respectively.

After stratification by gender, the AUC in male patients was 0.6529 (95% CI: 0.4863–0.8196), with an optimal cut-off value of 216.1 HU (sensitivity: 82.35%, specificity: 55.0%). Among female patients, the AUC was 0.6880 (95% CI: 0.5197–0.8563), with an optimal cut-off value of 163.4 HU (sensitivity: 41.30%, specificity: 70.59%). See Figure 3.

## 4 Discussion

This study measured femoral head HU values via CT to systematically investigate the relationship between local bone density and hip fracture type, stability, and sex. With the aging population, osteoporotic hip fractures bring heavy clinical and social burdens characterized by high mortality and disability rates (14). Although dual-energy x-ray absorptiometry (DEXA) is the gold standard for bone mineral density (BMD) measurement, it has limitations in hip assessment and cannot be applied emergently after trauma. By comparison, CT-derived HU values are widely validated to reflect local bone quality conveniently without additional radiation or economic burden for patients (10). A meta-analysis published by Suo et al. in 2020 also showed a statistically significant relationship between lower femoral neck bone density and an increased risk of ankle fractures in the elderly (15). Because HU values are easier to obtain and do not add extra medical costs for patients, this study quantitatively measured femoral head HU values via CT to systematically evaluate the complex relationship between local bone density and hip fracture type, stability, and sex. The main findings include: (1) femoral head HU values cannot effectively distinguish femoral neck fractures (FNF) from intertrochanteric fractures (IFF); (2) unstable IFF presents significantly lower HU values with high-specificity predictive performance; (3) significant sexual dimorphism exists in bone density, requiring gender-specific evaluation criteria. The following discussion emphasizes the clinical implications of these results, along with inherent limitations.

No significant difference in femoral head HU values was observed between FNF and IFF groups. Both fractures belong to low-energy fragility fractures in elderly patients, and decreased proximal femoral bone quality serves as a common pathological basis (16). This study suggests that the specific type of hip fracture may no longer be primarily determined by the average bone density of a single region, such as the femoral head, but rather depends more on the mechanical vector (direction, magnitude, and point of application) acting on the hip during a fall, as well as the local anatomical structure and microstructural characteristics of the proximal femur. Reduced bone density in patients reflects a decline in trabecular strength and connectivity, which, under traumatic loading, leads to more severe comminution and posteromedial cortical fractures, ultimately resulting in unstable fracture patterns. This finding is consistent with the research of other scholars (15, 17). Therefore, while the overall average HU value of the femoral head—as a component of the proximal femur—can reflect bone quality in that region, it cannot accurately predict subtle structural differences at specific “mechanical weak points” such as the femoral neck or intertrochanteric region. This suggests that when clinically assessing the risk of hip fractures, one should not rely solely on the average bone mineral density of a single site but should instead focus on the structural integrity and biomechanical characteristics of the proximal femur. Consistent with this notion, Kaya and Karabak recently demonstrated that proximal femoral morphometric features and radiographic bone quality indices were closely associated with hip fracture patterns in elderly patients. Traditional indicators such as Dorr classification, Singh index, and canal-to-bone ratio also contribute to evaluating femoral structural fragility and predicting fracture localization, which further supports the importance of comprehensive assessment beyond single-region bone mineral density (18).

The most clinically significant finding of this study is that, among patients with IFF, those with unstable fractures (AO/OTA 31-A2.2–A3) had significantly lower femoral head HU values than those with stable fractures (A1.1–A2.1). This association has an intuitive pathophysiological basis: a decrease in HU values directly reflects a reduction in bone mineral content within cancellous bone, as well as the thinning and fracturing of the trabecular network. This degradation of bone microstructure not only reduces the skeleton's ability to resist compression but, more critically, diminishes its capacity to withstand shear and torsional stresses. During low-energy trauma, this makes it more likely for comminuted fracture lines and the collapse of key supporting structures (such as the femoral neck) to occur, thereby forming radiographically defined unstable fracture patterns (19). ROC curve analysis further quantified this predictive value. Although the area under the curve (AUC) was 0.6764, indicating moderate overall discriminatory ability, specificity reached as high as 91.89% at the optimal cutoff value (143.5 HU). Notably, the optimal cutoff of 143.5 HU derived from the Youden index achieved extremely high specificity (91.89%) but low sensitivity (17.50%). This indicates that while a value below 143.5 HU strongly predicts an unstable fracture (low false-positive rate), approximately 82% of unstable fractures would be missed at this threshold. This sensitivity–specificity trade-off must be emphasized: HU values are highly specific for confirming unstable fractures but not sufficiently sensitive to be used as a standalone screening tool.

The low sensitivity reflects that unstable intertrochanteric fractures may occur across a range of bone densities, and bone quality is only one component of fracture stability. Therefore, HU values should be interpreted as an adjunctive imaging marker rather than a definitive screening test. When combined with clinical and morphological features, this high-specificity index remains valuable for preoperative risk stratification, particularly for identifying patients at the highest risk of instability. In other words, when a patient with an IFF has a femoral head HU value below 143.5 HU, there is a greater than 90% probability that the fracture is unstable. This provides critical guidance for preoperative planning: Surgical strategy and implant selection: A preoperative prediction of an unstable fracture prompts the surgical team to prioritize internal fixation options with superior biomechanical performance, such as conventional preparation long-length, head-shaft-integrated intramedullary nails (e.g., PFNA, InterTan), rather than dynamic hip screws (DHS), to better control rotational and axial instability and reduce the risk of fixation failure (20). More importantly, this measurement can be performed rapidly and non-invasively during the patient's initial CT scan upon admission. It is not only useful in patients with existing fractures but also extends its predictive value to community screening and fracture prevention. For elderly individuals without fractures who undergo abdominal or pelvic CT scans for other reasons (such as abdominal pain or routine physical examinations), measuring the femoral head HU value and detecting extremely low values (e.g., <150 HU) indicates severe osteoporosis and an extremely high risk of hip fracture, thereby prompting more proactive interventions with antiosporotic medications and fall prevention measures (21).

The gender differences observed in this study represent another key finding that warrants close attention. The mean HU values in male IFF patients were significantly higher than those in female patients, and this difference persisted in the unstable fracture subgroup. More critically, the ROC analysis revealed a marked disparity in the gender-specific optimal cutoff values (216.1 HU for men vs. 163.4 HU for women), with markedly different diagnostic performance patterns: the cutoff value for men exhibited high sensitivity (82.35%), making it suitable for screening; whereas the cutoff value for women demonstrated superior specificity (70.59%), making it suitable for confirmation. This “biphasic” phenomenon results from the interplay of complex physiological and pathological mechanisms. Men naturally possess higher peak bone mass, thicker cortical bone, and different proximal femoral geometry (such as a typically shorter hip axis), which results in higher absolute bone density values compared to women (22). In women, particularly postmenopausal women, bone resorption exceeds bone formation, leading not only to rapid bone mass loss but, more critically, to changes in trabecular microstructure. This qualitative damage is far more severe than a simple reduction in bone mass (23). In addition, the incidence of sarcopenia is higher in elderly women; the decline in muscle mass and strength weakens the ability to cushion and protect against falls and reduces mechanical loading on the skeleton, further exacerbating bone loss (24, 25). Consequently, female patients may experience unstable fractures even at “relatively high” bone density values (though still below male thresholds) due to severe deterioration of bone microstructure and insufficient muscular protection. This is clearly evidenced by the fact that the mean HU value (165.35 ± 32.98) in female patients with unstable fractures in this study was often lower than the CT thresholds for osteoporosis reported in many studies. This finding suggests that using a single bone density threshold (whether DEXA T-scores or CT HU values) to assess fracture risk or severity in all patients is crude and unscientific. It may lead to underestimation of risk in male patients or overemphasis on female patients. Therefore, it is essential to establish gender-, age-, and even race-specific bone density reference databases and diagnostic thresholds. When interpreting HU values, clinicians must make differentiated judgments based on the patient's gender.

This study found that although low HU values, female gender, and unstable fracture types are clearly associated with each other, these factors did not directly translate into poorer early radiographic outcomes (such as loss of reduction, cutting of internal fixation devices, or fractures) in a cohort of patients who underwent modern internal fixation surgery. This contrasts with the view held in a large body of historical literature, which identifies osteoporosis as a major risk factor for internal fixation failure (26–28). This may precisely reflect advances in modern orthopedic surgical concepts and techniques. With the widespread use of locking plate systems, the incidence of complications such as screw extrusion in osteoporotic fractures has decreased. For areas with severe bone defects, intraoperative bone grafting using allograft, autograft, or bone substitute materials is performed to enhance the initial stability of internal fixation and promote bone healing (29). Identifying high-risk patients with low HU values preoperatively not only guides the surgical plan but should also trigger a management protocol spanning the perioperative period and beyond, including the early postoperative use (typically within one week) of antiresorptive or anabolic agents, supplementation with calcium and vitamin D, and standardized rehabilitation training (30).

This study has several limitations. Firstly, the retrospective, single-centre design may introduce selection bias, and unmeasured confounders (such as detailed medication history or fall mechanisms) could exert influence. Secondly, the sample size may have limited the statistical power of subgroup analyses, and the generalisability of the derived HU cutoff values across different CT equipment requires further validation. Fourthly, the primary outcome being radiographic stability, the absence of patient-centred functional outcomes or long-term follow-up data necessitates cautious interpretation of inferences regarding surgical outcomes independent of bone quality. Fifth, the optimal HU cutoff showed a substantial sensitivity–specificity trade-off with low sensitivity for detecting unstable fractures, limiting its utility as a standalone screening tool. Finally, the model excluded potential predictors such as muscle cross-sectional area or more detailed bone microstructural analysis, which may enhance predictive performance in future models.

## 5 Conclusions

Although the HU value of the femoral head cannot effectively distinguish between femoral neck fractures and intertrochanteric fractures, it serves as a significant radiographic marker for predicting the stability of intertrochanteric fractures, exhibiting high specificity (91.89%) and being suitable for risk indication. Patients with unstable fractures exhibited significantly lower HU values than those with stable fractures. ROC curve analysis indicated this metric possesses high specificity (91.89%), proving particularly valuable for confirming stable fracture status. Crucially, this study first demonstrated significant gender-specific differences in this predictive indicator through ROC analysis. Male and female patients require distinct optimal diagnostic cut-off values (216.1 HU for males, 163.4 HU for females), with differing diagnostic performance patterns, strongly indicating the necessity for establishing gender-specific assessment criteria. Although low bone density correlates with unstable fractures, modern internal fixation and bone grafting techniques can effectively improve early prognosis. Future efforts should focus on developing integrated multi-factor models incorporating HU values to achieve more precise individualised preoperative planning.

## Data Availability

The original contributions presented in the study are included in the article/Supplementary Material, further inquiries can be directed to the corresponding author.
